# Unusual branching pattern and termination of facial artery and its clinical implications for facial operations

**DOI:** 10.1590/1677-5449.190021

**Published:** 2019-07-12

**Authors:** Ashwini Aithal Padur, Naveen Kumar

**Affiliations:** 1 Manipal Academy of Higher Education (MAHE), Melaka Manipal Medical College (Manipal campus), Department of Anatomy, Manipal, Karnataka, India.

**Keywords:** facial artery, transverse facial artery, face, facial surgery, artéria facial, artéria facial transversa, face, cirurgia da face

## Abstract

The facial artery is the main artery of the face and variations in its origin and its branching pattern have been documented. We report herein multiple facial artery branch variations in the face. A large posterior (premasseteric) branch originated from the left facial artery and coursed upwards behind the main trunk of the facial artery. This artery presented with a straight course and was closely related to the anterior border of the masseter. The branch then terminated by supplying the adjacent connective tissue below the parotid duct. It was also observed that the facial artery was very thick and tortuous and terminated as the superior labial artery. Knowledge of this variation is of great clinical significance in facial operations, especially for maxillofacial surgeons and plastic surgeons, because it forms the anatomical basis for the facial artery musculo-mucosal flap.

## Resumo

A artéria facial é a principal artéria da face, e variações da sua origem e padrão de ramificação têm sido documentadas. Este artigo descreve múltiplas variações de ramificações da artéria facial. Um ramo posterior grande (pré-massetérico) teve origem na artéria facial esquerda e cursou para cima por trás do tronco principal da artéria facial. Essa artéria apresentou um curso reto e intimamente relacionado com a borda anterior do masseter. O ramo terminou suprindo o tecido conjuntivo adjacente abaixo do ducto parotídeo. Também se observou que a artéria facial tinha grande calibre, era tortuosa e terminava na artéria labial superior. Conhecer essa variação é de grande significância clínica em cirurgias da face, principalmente para cirurgiões maxilofaciais e plásticos, tendo em vista que ela forma a base anatômica para o retalho músculo-mucoso da artéria facial.

## INTRODUCTION

The face is supplied by branches of the facial artery and the superficial temporal arteries. The facial artery originates in the neck, arising from the external carotid artery, and terminates at the medial angle of the eye. This artery’s tortuosity allows it to stretch during various movements of the jaw. The branches of the facial artery in the face are the superior labial artery, inferior labial artery, and the lateral nasal artery, which supply the muscles and skin of the face. It also gives off a few small unnamed branches posteriorly.

The facial artery presents variations with regards to its origin, termination, course, and branching pattern. We report herein a variant termination of the facial artery and the occurrence of a large posterior branch of the facial artery known as the premasseteric artery. This is a minor inconsistent branch,^1^ which passes upwards in the face. Knowledge regarding any variations of the facial artery may turn out to be vital during maxillofacial, orofacial, and rhinoplastic surgeries. It is also important during execution of some surgical procedures, such as the facial artery musculo-mucosal flap,^2^ used for reconstruction of oronasal fistulas.^3^

## CASE REPORT

**Figure 1:**
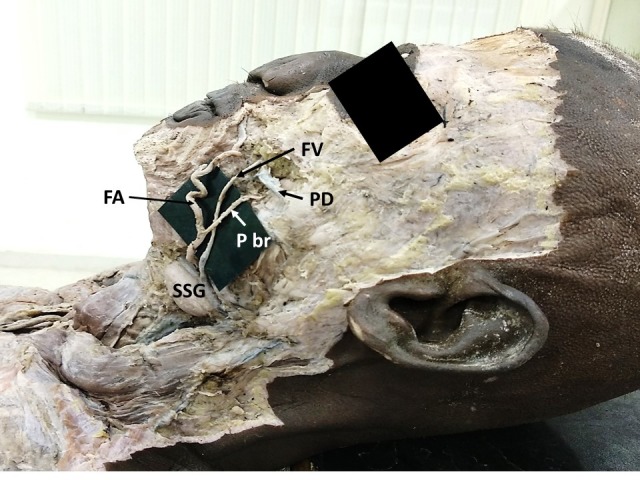
Figure showing the origin of the premasseteric branch (P br) from the facial artery (FA). FV = facial vein; PD = parotid duct; SSG = submandibular salivary gland.

**Figure 2:**
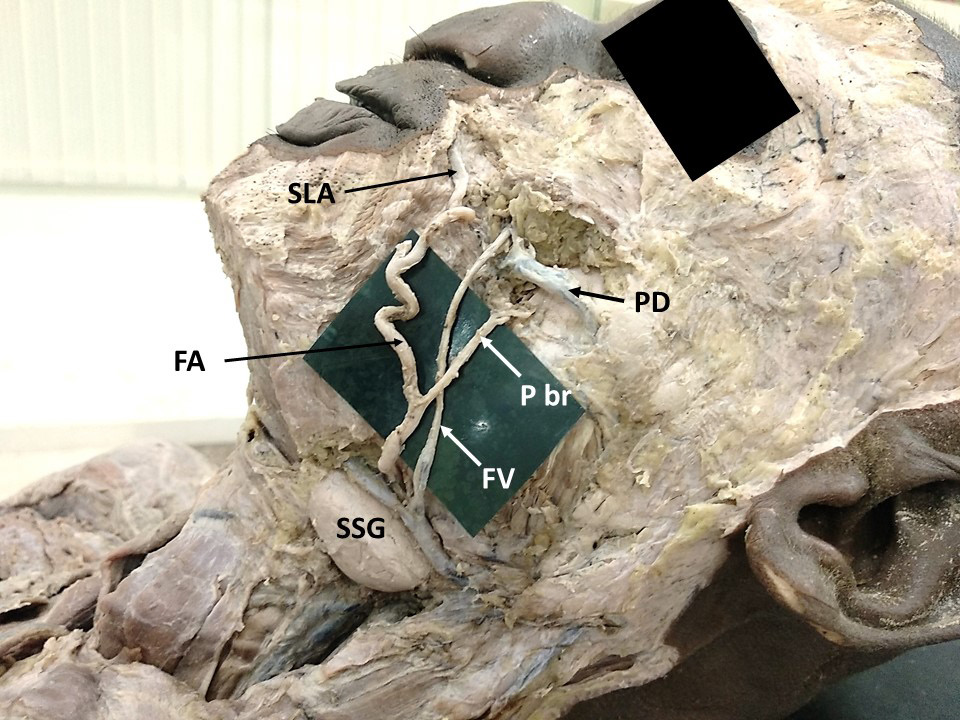
Closer view showing the premasseteric branch (P br) and termination of the facial artery (FA) as the superior labial artery (SLA). FV = facial vein; PD = parotid duct; SSG = submandibular salivary gland.

During dissections for undergraduate medical students, we found unusual variations in the branching pattern and termination of the left facial artery in the face. These variations were observed in a male cadaver aged about 65-70 years. The left facial artery presented a large posterior branch (premasseteric branch) which coursed upwards parallel to the facial artery (Figure 1). After its origin, this premasseteric branch ran upwards and crossed the facial vein superficially, from medial to lateral. It ran superficial to the cheek muscles, and anterior to the masseter muscle. It then terminated near the parotid duct, supplying the surrounding tissues. After giving off this posterior branch, the main trunk of the facial artery ran on the lateral side of the lower lip and gave off an inferior labial branch. Then it coursed tortuously lateral to the angle of mouth and terminated at the upper lip as the superior labial artery (Figure 2). Hence, the lateral nasal branch and angular branch were absent. Branches of the infraorbital artery supplied the lower eyelids and lateral part of the nose.

## DISCUSSION

Variations related to the facial artery or its branches have two aspects of interest: In operations of the face and lip, during reconstructive and reparative procedures, and in radiologic anatomy, in the field of malignancy for the treatment of some facial tumors by embolization. There are reports in the literature of variations in the origin, termination, and branches of the facial artery. The premasseteric branch is an uncommon branch that was first described by Adachi in 1928.^4^ If present, this branch can be expected to be injured during maxillofacial surgeries, causing severe hemorrhage. Kumar et al. stated that during its course the premasseteric branch could compress some structures such as the facial vein and the parotid duct.^5^ The masseter is a key muscle which is frequently exploited by craniofacial surgeons in operations to correct facial palsy and benign masseteric hypertrophy, or neurectomy-induced muscle atrophy. Thus, understanding of this premasseteric branch and its relations with the adjacent structures in the face is crucial for maneuvering the masseter muscle safely and avoiding complications in such procedures.

Bayram et al. has studied variations of the facial artery in fetuses.^6^ Based on these observations, he categorized the variations into: Type I category – facial artery terminated as angular artery, Type II category – facial artery terminated as superior labial artery, and Type III category – facial artery terminated as inferior labial artery. In his study, Type I facial artery was found in 76% of hemi-faces, Type II in 12% and Type III in 12% of hemi-faces. In our present report, a Type II variation was observed in which the facial artery terminated as the superior labial artery. The incidence of this particular variation is said to range from 4%^7^ to 8.4%.^8^

The face is richly vascularized and so construction of several facial flaps is possible. Reconstruction of lip defects using procedures like the Abbe flap and other lip flap procedures involves surgical manipulation of one of the major facial artery branches, mainly the superior labial artery.^8^ The facial artery musculo-mucosal (FAMM) flap is a recent technique with many advantages, but its use is restricted due to vast variations in the course of the facial artery. The facial artery is also carefully chosen as a target for intra-arterial chemotherapy in treatments for some cancers of the head.^9^ Therefore, precise knowledge regarding the detailed anatomy of the facial artery is of utmost importance.

Maxillofacial and plastic surgeons must have detailed knowledge of such variations before deciding on any grafts or surgical interventions on the face. Knowledge regarding such variations is also imperative for general surgical practitioners and specialists, for the effective management of any injuries, or correction of congenital anomalies like cleft lip and palate. Such anomalous variations therefore warrant use of a noninvasive in vivo technique for evaluation of the facial artery anatomy in order to facilitate preoperative planning in complex facial reconstructions.

## References

[1] Standring S (2005). Gray’s Anatomy: the anatomical basis of clinical practice..

[2] Pribaz J, Stephens W, Crespo L, Gifford G (1992). A new intra oral flap: facial artery musculomucosal (FAMM) flap. Plast Reconstr Surg.

[3] Dupoirieux L, Plane L, Gard C, Penneau M (1999). Anatomical basis and results of the facial artery musculomucosal flap for oral reconstruction. Br J Oral Maxillofac Surg.

[4] Adachi B. (1928). Arteriensystem der Japaner.

[5] Kumar N, Nayak SB, Shetty S, Guru A (2011). Unusual posterior (premasseteric) branch of facial artery – a case report. IJAV.

[6] Bayram SB, Kalaycioglu A (2010). Branching patterns of facial artery in fetuses. N J Med.

[7] Niranjan NS (1988). An anatomical study of the facial artery. Ann Plast Surg.

[8] Loukas M, Hullet J, Louis RG (2006). A detailed observation of variations of the facial artery, with emphasis on the superior labial artery. Surg Radiol Anat.

[9] Shimizu T, Sakakura Y, Hattori T, Yamaguchi N, Kubo M, Sakakura K (1990). Super selective intraarterial chemotherapy in combination with irradiation: preliminary report. Am J Otolaryngol.

